# MicroRNA Expression Profiling to Identify and Validate Reference Genes for the Relative Quantification of microRNA in Rectal Cancer

**DOI:** 10.1371/journal.pone.0150593

**Published:** 2016-03-03

**Authors:** Anne Haahr Mellergaard Eriksen, Rikke Fredslund Andersen, Niels Pallisgaard, Flemming Brandt Sørensen, Anders Jakobsen, Torben Frøstrup Hansen

**Affiliations:** 1 Danish Colorectal Cancer Center South, Center of Clinical Excellence, Vejle Hospital, Vejle, Denmark; 2 Institute of Regional Health Research, University of Southern Denmark, Odense, Denmark; 3 Department of Clinical Pathology, Roskilde Hospital, Roskilde, Denmark; National Institutes of Health, UNITED STATES

## Abstract

**Introduction:**

MicroRNAs (miRNAs) play important roles in regulating biological processes at the post-transcriptional level. Deregulation of miRNAs has been observed in cancer, and miRNAs are being investigated as potential biomarkers regarding diagnosis, prognosis and prediction in cancer management. Real-time quantitative polymerase chain reaction (RT-qPCR) is commonly used, when measuring miRNA expression. Appropriate normalisation of RT-qPCR data is important to ensure reliable results. The aim of the present study was to identify stably expressed miRNAs applicable as normaliser candidates in future studies of miRNA expression in rectal cancer.

**Materials and Methods:**

We performed high-throughput miRNA profiling (OpenArray®) on ten pairs of laser micro-dissected rectal cancer tissue and adjacent stroma. A global mean expression normalisation strategy was applied to identify the most stably expressed miRNAs for subsequent validation. In the first validation experiment, a panel of miRNAs were analysed on 25 pairs of micro dissected rectal cancer tissue and adjacent stroma. Subsequently, the same miRNAs were analysed in 28 pairs of rectal cancer tissue and normal rectal mucosa.

**Results:**

From the miRNA profiling experiment, miR-645, miR-193a-5p, miR-27a and let-7g were identified as stably expressed, both in malignant and stromal tissue. In addition, NormFinder confirmed high expression stability for the four miRNAs. In the RT-qPCR based validation experiments, no significant difference between tumour and stroma/normal rectal mucosa was detected for the mean of the normaliser candidates miR-27a, miR-193a-5p and let-7g (first validation *P* = 0.801, second validation *P* = 0.321). MiR-645 was excluded from the data analysis, because it was undetected in 35 of 50 samples (first validation) and in 24 of 56 samples (second validation), respectively. Significant difference in expression level of RNU6B was observed between tumour and adjacent stromal (first validation), and between tumour and normal rectal mucosa (second validation).

**Conclusion:**

We recommend the mean expression of miR-27a, miR-193a-5p and let-7g as normalisation factor, when performing miRNA expression analyses by RT-qPCR on rectal cancer tissue.

## Introduction

MicroRNAs (miRNAs) are short non-coding RNA molecules acting as negative gene regulators at the post-transcriptional level. These small RNAs consist of 18–25 nucleotides and play an important role in the regulation of biological processes such as cell differentiation, proliferation, and apoptosis. Changes in miRNA expression profiles are associated with abnormal cellular functions and have been observed in a variety of diseases, including cancer [1, 2]. Many miRNAs target oncogenes and tumour suppressor genes with direct involvement in carcinogenesis [3]. Because of the association between many miRNAs and cancer, miRNAs are being investigated as potential biomarkers regarding diagnosis, prognosis and prediction in cancer management [2, 4].

Real-time quantitative polymerase chain reaction (RT-qPCR) is widely accepted as the method of choice for relative gene expression quantification, since it is the most sensitive and reproducible method. However, the accuracy and precision of the results are dependent on proper data normalisation. Reference genes are necessary to correct for non-biological sample-to-sample variations, which could be introduced during all steps from sample preparation to amplification. The use of non-stably expressed reference genes for normalisation may lead to incorrect conclusions. It is generally accepted that a universal reference gene suitable for all tissue types does not exist [5–8]. The most accurate approach is to select stably expressed reference genes for each specific experiment according to the tissue of interest [9].

Despite an increasing number of miRNA expression studies, the literature is extremely sparse, when it comes to rectal cancer and reliable normaliser candidates.

The aim of the present study was to identify stably expressed miRNAs in rectal cancer tissue to be used as normalisers in future miRNA expression studies. In this context miRNA-normalisers will be referred to as reference genes. We studied the expression profile of miRNAs in laser micro-dissected tumour tissue and adjacent stromal tissue from specimens of patients with rectal cancer. First, we selected the most stably expressed miRNAs from our high-throughput technology based miRNA expression profiling study on 10 patients (OpenArray®, Life Technologies). Secondly, the stability of these miRNAs was further assessed by RT-qPCR in rectal cancer tissue from 25 other patients. Finally, we analysed the stability of the same miRNAs in rectal cancer tissue and normal rectal mucosa from 28 comparable patients. We included the commonly used small nuclear RNA RNU6B in all our analyses.

## Materials and Methods

### Patients and tissue samples

Patients were randomly selected from a cohort of 239 patients undergoing resection of rectal cancer at Vejle Hospital, Denmark from 1999 to 2008. Inclusion was accomplished only if their histopathological diagnosis were rectal adenocarcinoma, if they had no preoperative chemo-radiotherapy, and if formalin-fixed paraffin-embedded (FFPE) rectal cancer tissue was available.

For the miRNA expression profiling analysis, tumour tissue from a total of 10 patients (5 male, 5 female; median age 74, range 59–86 years) was selected representing different pathological tumor stages (pT1-pT4) and different amount of lymphocytes in the tumour micro-environment (Table 1). For the first validation study, tissue from another 25 rectal cancer patients (13 male, 12 female; median age 68, range 49–87 years) was selected according to tumour stage, but regardless of the number of stromal lymphocytes; fifteen tumours were classified pT3, ten tumours were classified pT4. For the second validation study, rectal cancer tissue and normal rectal mucosa from 28 other patients (13 male, 15 female; median age 70, range 45–89 years) were included; all tumours were classified pT3.

**Table 1:** Characteristics of rectal cancer specimens for miRNA expression profiling analyses.

Pathological Tumour Stage	Number with High Amount of Lymphocytes*	Number with Low Amount of Lymphocytes*	Total
**pT1**	1	1	2
**pT2**	1	1	2
**pT3**	2	2	4
**pT4**	1	1	2

*The amount of lymphocytes in the tumour micro-environment was qualitatively scored by a pathologist (FBS). A high amount represents a continuous, broad lymphocytic infiltration along the invasive tumour front, often with follicular aggregation. A low amount represents a patchy, sparse lymphocytic infiltration along the invasive tumour front.

According to The Danish National Committee on Health Research Ethics, ethical approval and written informed consent were not required, because the purpose of the study was quality development and no clinical data were further analysed (Act on Research Ethics Review of Health Research Projects, cf. § 14, section 1). No samples were collected specifically for the purpose of this study. The samples were collected during standard treatment. The project was done anonymously. The tissue used in this study was confirmed not to be included in the Danish Registry of Human Tissue Utilisation. The study has been reported to the Danish Data Protection Agency.

### Laser micro-dissection

**Fig 1:**
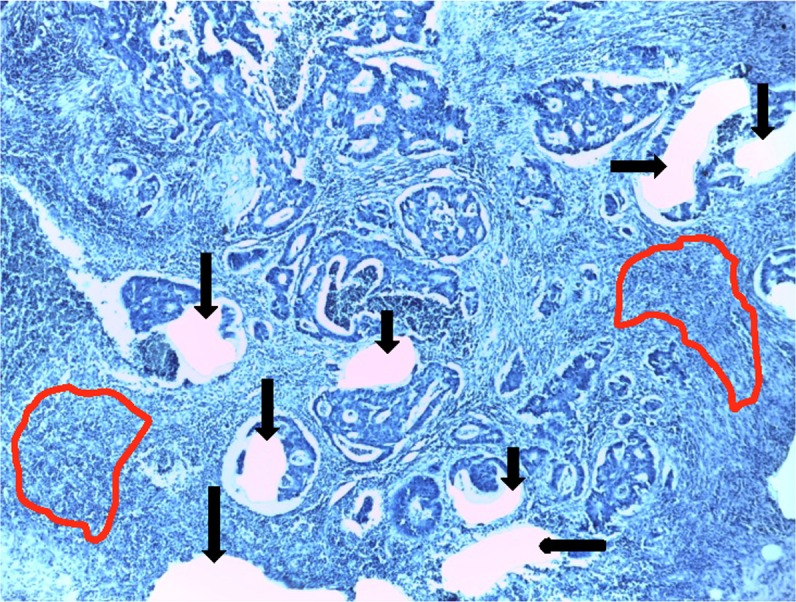
Laser micro-dissection. Areas of tumour cells have been removed (arrows). Areas to be removed from the stromal tissue have been marked (red lines).

To specifically evaluate miRNA expression in rectal adenocarcinoma cells and in the surrounding stroma, laser micro-dissection (LMD) was performed (Fig 1). Available histological sections stained with hematoxylin and eosin were examined by a pathologist in order to select the deepest invasive FFPE section from the tumour. For the miRNA expression profiling, an equal number of cases with a high versus a low amount of lymphocytes in the adjacent stromal tissue were selected (Table 1). According to the above selection, sections (8–10 μm) were cut from FFPE tissue and affixed to frame slides for membrane-based LMD, using Leica LMD6500 Microsystems (Leica). Slides were dried for 45 min at 60°C, washed two times for 5 min in xylene to remove paraffin, rehydrated by a graded ethanol series (99%, 99%, 96%, 70%), rinsed in demineralised water, stained for 3 min with hematoxylin, rinsed for 5 min in demineralised water, and allowed to air-dry. Tumour cells (a) and tumour micro-environmental stroma (b) were isolated by LMD and separately collected in the caps of 0.5 ml RNase-free PCR tubes, where a drop of ethanol was added. For the second validation experiment, the tumour cells were isolated by LMD, whereas the normal rectal mucosa was removed from the slides with a scalpel.

### RNA extraction

RNA was isolated using the miRNeasy FFPE Kit (Qiagen, Hilden, Germany) according to the manufacturer’s instructions. Since paraffin was removed before LMD, first step was adding a lysis buffer containing proteinase K, followed by a short incubation at a higher temperature. This was followed by DNase treatment and RBC buffer treatment. Ethanol was added and the sample was applied to an RNeasy MinElute spin column, where the total RNA, including miRNA, bound to the membrane was washed away. Finally, total RNA, including miRNA, was eluted in 14 μl RNase-free water.

### OpenArray® Panels

OpenArray® Human MicroRNA Panel (Life Technologies, Foster City, CA, USA) is a high-throughput PCR-based miRNA array that enables analysis of more than 750 pre-defined miRNAs on a microfluidic platform. Single-stranded cDNA was reverse transcribed from total RNA using Megaplex™ RT Primers, Human Pool A or B. Each RT reaction had a final volume of 7.5 μl and contained total RNA (3 μl) and reagents from TaqMan® MicroRNA Reverse Transcription Kit (4.5 μl) (Life Technologies).

Preamplification (16 cycles) was performed with Megaplex™ PreAmp Primers, Human Pool A or B (Life Technologies) and TaqMan PreAmp Mastermix (Life Technologies) according to the manufacturer’s standard instructions for low sample input (LSI). The PreAmp Product was diluted 1:40 in TE-buffer. To test the result of reverse transcription and preamplification before we proceeded to OpenArray®, the products were tested with single assay qPCR for either miR-16 (Pool A) or miR-151-3p (Pool B). Analyses were done in 20 μl reactions in duplicate using 1.3 μl of diluted sample. Both miRNAs were detected.

For the qPCR step, TaqMan® OpenArray® Human MicroRNA Panels (Life Technologies) were used according to the manufacturer’s instructions. qPCR was performed using QuantStudio 12K Flex System (Life Technologies). All reactions were performed in triplicate, where each sample was analysed on three different OpenArray® Panels.

### Validation RT-qPCR

The Custom TaqMan® Array MicroRNA Cards (Life Technologies) were used according to the manufacturer’s standard instructions for LSI. RNA was reverse-transcribed using Custom MiRNA RT Primer Pools supplied together with the Array Cards. Each RT reaction contained 3 μl total RNA and 4.5 μl RT reaction mix from TaqMan® MicroRNA Reverse Transcription Kit.

5 μl RT product was preamplified (14 cycles) with Custom PreAmp Primer Pools (supplied with the Array Cards) and TaqMan PreAmp Mastermix in 25 μl reactions. The PreAmp Product was diluted 1:4 in TE-buffer. qPCR analyses were carried out using TaqMan® Universal Master Mix II NoAmpErase® UNG and the Applied Biosystems 7900 HT Real-Time PCR System. All reactions were performed in triplicate.

Both validation experiments followed the same steps as described above.

### Data analysis

High throughput data generated from TaqMan® OpenArray® RT-qPCR was analysed using Expression Suite Software Version 1.0.3 (Life Technologies), a data-analysis tool that utilises the comparative Cq (ΔΔCq) method to quantify relative gene expression across a large number of genes and samples. Relative quantification (RQ)–also called Fold Change (FC)—was calculated from Cq values according to the equations: (a) ΔCq = Cq (miRNA)–Cq (global mean); (b) ΔΔCq = ΔCq (tumour)–ΔCq (stroma); and (c) RQ = 2^−ΔΔ*Cq*^, where Cq is defined as the PCR cycle number at which the fluorescence meets the threshold in the amplification plot.

Expression Suite performs an unpaired t-test for biological group comparisons, assuming that Cq values for both groups follow a normal distribution.

To analyse the expression stability of the reference gene candidates, the software program NormFinder [10] was used according to the developer’s recommendations. NormFinder calculates a stability value for a panel of reference gene candidates combining the inter- and intra-group expression variation. The lowest values indicate the most stably expressed reference genes. Average values of triplicate Cq values were converted to linear values for Norm Finder and statistical analyses, by the equation linear value (Cq) = 2^−*Cq*^, assuming that the amplification efficiency for the reference gene assays is close to 100%. Statistical analyses were performed using NCSS 2007 (NSCC LLC, Kaysville, UT, USA). In the validation experiments, Wilcoxon Rank-Sum Test was used to test for differences in medians, when comparing tumour and stroma (first validation) and tumour and normal mucosa (second validation). P-values below 0.05 were considered statistically significant for all tests.

## Results

### Identification of candidate reference genes using Expression Suite Software

We profiled a panel of more than 750 miRNAs and controls on 10 pairs of rectal cancer tissue and adjacent stromal tissue. Initially, a global mean expression normalisation strategy was applied to identify the most stably expressed miRNAs [2]. Data were excluded from further analyses, if Cq-values exceeded 35 and/or the amplification score was below 1.24.

From the Expression Suite data-analysis, the four reference gene candidates let-7g, miR-193-5p, miR-27a, and miR-645 (Table 2) were selected from the following criteria: (a) Relative quantification (RQ) close to 1:1 (where the reference biological group is stromal tissue); (b) miRNA detected in more than 70% of all replicates of all samples; (c) Cq values between 18 and 28; (d) No intentions of including the miRNA as a target in our future projects. In addition, we looked at the p-value, considering a high p-value was suggesting no major difference between the tissue groups and no major spread of Cq values within the groups.

**Table 2:** Selected reference gene candidates from the Expression Suite data-analysis.

miRNA	RQ*	Detection**	Cq(mean), tumour/stroma	P-value
**miR-645**	1:1.09	76%	21.2 / 22.0	0.809
**let-7g**	1:1.11	82%	23.0 / 24.1	0.873
**miR-193a-5p**	1:1.14	84%	24.5 / 26.4	0.825
**miR-27a**	1:1.15	98%	19.7 / 21.2	0.997

* RQ = relative quantification. Reference biological group (= 1) is stromal tissue

** Percentage of samples where the miRNA was detected

To add another generally accepted approach, a panel of 10 miRNAs, all meeting the above mentioned criteria ((a)-(d)), were analysed using Norm Finder (Table 3). RQ ranged from 1:0.8 to 1:1.2 (where the reference biological group was stroma). The p-value was not considered when selecting the miRNAs for this analysis. RNU6B was included in this analysis. The investigation identified let-7g, miR-193a-5p, miR-27a, and miR-645 among a group of miRNAs with a low and almost equal stability value, indicating high expression stability. RNU6B showed a 10-times higher stability value, indicating lower expression stability than the other reference gene candidates. Changing from two biological groups (tumour and stroma) to three groups (tumour, stroma with low amount of lymphocytes, and stroma with high amount of lymphocytes) had only minor effect on the stability values (data not shown).

**Table 3:** Stability values for reference gene candidates according to Norm Finder.

Reference Gene	Stability Value
**miR-99b**	0.00002
**miR-27a**	0.00002
**miR26b**	0.00002
**miR328**	0.00002
**miR645**	0.00002
**let-7g**	0.00002
**miR-193-5p**	0.00002
**miR-548c-5p**	0.00002
**miR-548d-5p**	0.00002
**miR-25**	0.00002
**RNU6B**	0.00023

Analyses performed on two groups of biological samples (tumour and stroma). The lowest stability value indicates the highest stability. The reference gene candidates selected based on the Expression Suite analyses are let-7g, miR-193a-5p, miR-27a, and miR-645.

### RT-qPCR-based validation of reference gene candidates in tumour and adjacent stroma

**Fig 2:**
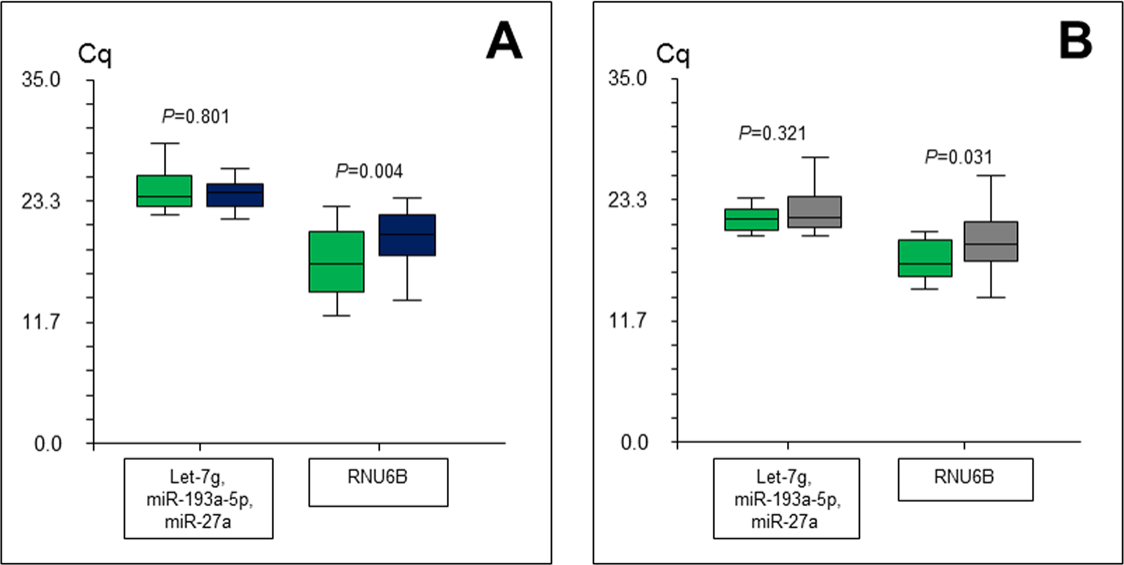
**Expression of reference gene candidates in rectal cancer tissue (n = 25) and adjacent stromal tissue (n = 25) (A), and in rectal cancer tissue (n = 28) and normal rectal mucosa (n = 28) (B).** Values are given as quantification cycles (Cq). Boxes (green, cancer tissue; blue, stromal tissue; grey, normal mucosa) represent upper and lower quartiles with medians as horizontal lines. Whiskers depict 1.5 x the interquartile range. Outliers are not shown. P-values are according to Wilcoxon Rank Sum Test for difference in medians. RNU6B showed a significant difference between tumour and stroma (A), and between tumour and normal mucosa (B). There is no significant difference in the mean expression level of let-7g, miR-193a-5p and miR-27a between tumour and stroma (A), and between tumour and normal mucosa (B).

The expression patterns of let-7g, miR-193a-5p, miR-27a, miR-645 and RNU6B were further analysed on 25 pairs of rectal cancer and adjacent stromal tissue. Because miR-645 was only detected in 15 of 50 samples, this miRNA was eliminated from further data analyses. Let-7g, miR-193a-5p and miR-27a showed different but measurable expression levels and all of them were expressed in all 50 samples. Raw Cq levels ranged from 20.1 to 28.9 for let-7g, from 23.2 to 31.1 for miR-193a-5p, and from 19.7 to 26.4 for miR-27a. For RNU6B the raw Cq level ranged from 12.0 to 23.6. Box plots are shown for the arithmetic mean of let-7g, miR-193a-5p and miR-27a, and for RNU6B in Fig 2. The arithmetic mean of the Cq values for let-7g, miR-193a-5p and miR-27a, and the Cq values for RNU6B were converted into linear values, and Wilcoxon Rank-Sum Test for difference in medians was performed. We observed a significant difference for RNU6B (*P* = 0.004) between the tumour tissue and the adjacent stromal tissue, whereas no significant difference was detected for the mean of the other three reference gene candidates: let-7g, miR-193a-5p and miR-27a (*P* = 0.801).

### RT-qPCR-based validation of reference gene candidates in tumour and normal mucosa

As a second validation, the expression patterns of let-7g, miR-193a-5p, miR-27a, miR-645 and RNU6B were analysed in 28 pairs of rectal cancer tissue and normal rectal mucosa. MiR-193a-5p, miR-27a and RNU6B were detected in all 56 samples. Let-7g was detected in 55 samples and undetected in one sample with normal rectal mucosa. Because miR-645 was only detected in 32 of 56 samples, this miRNA was not included in the further data analysis. Raw Cq levels ranged from 18.2 to 29.5 for let-7g, from 20.9 to 34.7 for miR-193a-5p, from 18.7 to 29.5 for mi-27a, and from 14.3 to 29.7 for RNU6B. Box plots are shown in Fig 2. No significant difference was detected for the arithmetic mean of let-7g, miR-193a-5p and miR-27a (*P* = 0.321) between the tumour tissue and the normal mucosa, whereas for RNU6B, we observed a significant difference (*P* = 0.031).

## Discussion

The use of reliable reference genes for normalisation is of great importance, when performing RT-qPCR based research. The normalisation procedure compensates for variations in RNA-extraction yield, reverse-transcription yield, and efficiency of amplification, thereby enabling comparison of miRNA expression levels across different samples [11].

It is widely accepted, that a single, universal reference gene does not exist, and it has been argued, that the most precise normalisation is achieved by selecting reference genes belonging to the same class of RNA as the investigated genes. Furthermore, it is recommended to search for stably expressed genes in each experimental system [12, 13].

Davoren *et al*. reported the first systematic identification of reference gene candidates for miRNA RT-qPCR experiments in breast cancer in 2008 [5]. In 2010, Chang *et al*. reported their identification of reference gene candidates for miRNA RT-qPCR in colorectal cancer [6], where they identified the top six most stably expressed miRNAs as let-7a, miR-16, miR-26a, miR-345, miR-425 and miR-454.

To find particular information about reference genes for miRNA expression studies in rectal cancer, we performed a PubMed search. Using the MeSH terms “MicroRNAs” and “Rectal cancer”, we found 9 articles on rectal cancer expression studies, published until February 2015 that included information on the use of reference genes (Table 4; [3, 14–21]).

**Table 4:** Reference genes used for normalisation of microRNA expression analyses in rectal cancer.

Reference gene(s)	Number of studies	References
RNU6B or U6	5	[3, 14–17]
U6 + U47	2	[20, 21]
RNU48	1	[18]
RNU44 + RNU48 + RNU66	1	[19]
let-7g + miR-27a + miR-193a-5p	Present study	

This search revealed that small nuclear RNAs were usual reference genes in rectal cancer experiments, where RNU6B was preferred. However using these small RNAs as reference genes can be problematic, despite they show stable expression levels in normal tissue, they have shown different expression levels in cancer tissue compared to normal tissue [4]. In addition, details of selection and validation of the used reference genes and whether or not they were stably expressed across the investigated tissue are often not provided. None of the studies applied a miRNA as reference gene.

Moreover, according to Peltier *et al*., the most commonly used reference gene in rectal cancer experiments, RNU6B, was demonstrated to be one of the least stably expressed RNA species [8].

To the best of our knowledge, this is the first OpenArray® based and subsequent RT-qPCR-validated identification of appropriate reference genes in rectal cancer specimens alone. Because, so far, RNU6B has been the most commonly used endogenous control in rectal cancer studies, we decided to include it in our experiments for comparison. We profiled the expression of human miRNAs (including RNU6B) on 10 pairs of rectal cancer tissue and adjacent stromal tissue. Since the reference genes are going to be used for normalisation in future RT-qPCR analyses on rectal cancer biopsies containing only tumour cells and adjacent stromal tissue, we did not include normal rectal mucosa in the initial analyses. By applying the global mean expression value for normalisation, we identified the most stably expressed miRNAs: let-7g, miR-193a-5p, miR-27a and miR-645. Global mean normalisation has previously been demonstrated to be superior to other approaches of normalisation in reducing technical variations and showing biological changes [2].

Subsequent use of NormFinder confirmed high expression stability for the four miRNAs, which were all about ten times more stable than the often used reference gene RNU6B. Since the amount of stromal lymphocytes did no substantial alterations to the stability values, we concluded that the amount of lymphocytes did not influence the expression levels of these miRNAs, and the number of lymphocytes in the stromal compartment was not taken into consideration in the validation experiments.

Our first validation experiment was carried out in a larger cohort of 25 pairs of tissue and confirmed no significant difference (*P* = 0.801) in mean expression value for let-7g, miR-193a-5p and miR-27a between rectal cancer tissue and adjacent stromal tissue. The absence of a significant difference does not prove an equal expression level between tumour tissue and adjacent stromal tissue; however, on the other hand a high p-value speaks in favour of equality or minor differences. The significant difference in expression of RNU6B between tumour tissue and adjacent stroma (*P* = 0.004) is in concordance with previous results [8]. MiR-645 was excluded, because it was not represented in all samples.

In order to further validate the results, we did a second validation in a cohort of 28 pairs of rectal cancer tissue and normal rectal mucosa. As in the two initial experiments, we did not find a significant difference (*P* = 0.321) between the arithmetic mean of the expression levels of let-7g, miR-193a-5p and miR-27a in the rectal cancer tissue and the compared compartment (in this case the normal rectal mucosa), whereas a significant difference was detected for the expression level of RNU6B (*P* = 0.031). As in the first validation study, miR-645 was undetected in a considerable part of the samples (24 of 56), and therefore still not suitable as a reference gene in this setting.

MiR-645 was broadly detected in OpenArray®, but not in the two validation experiments. Because there were no differences in the basic characteristics between the samples used in the identification experiment and the subsequent validations, the differences in detection rate is likely to be caused by the platform. The major variations in detection of miR-645 in in the different platforms emphasises the importance of validating results from a high-throughput method.

According to www.miRBase.org, let-7g, miR-193a-5p and miR-27a do not belong to the same miRNA-families, which reduce the chance that they might be co-regulated. The selection of three reference genes meet Vandesompele *et al*.’s recommendation to use at least three proper reference genes for calculating a normalisation factor [12].

In conclusion, based on a high-throughput RT-qPCR miRNA profiling study and two subsequent RT-qPCR based validation experiments, we recommend the mean value of let-7g, miR-193a-5p and miR-27a as normalisation factor, when performing miRNA expression analyses by RT-qPCR on rectal cancer tissue.

## Supporting Information

S1 Table Data from the initial profiling experiment (OpenArray®). (XLSX)

S2 Table Data from the first validation experiment (tumour and stroma). (XLSX)

S3 Table Data from the second validation experiment (tumour and normal rectal mucosa). (XLSX)
